# Complete Genome Sequences of Two Closely Related Paenarthrobacter nicotinovorans Strains

**DOI:** 10.1128/mra.00133-22

**Published:** 2022-05-10

**Authors:** Amada El-Sabeh, Iasmina Honceriu, Fakhri Kallabi, Razvan-Stefan Boiangiu, Marius Mihasan

**Affiliations:** a Faculty of Biology, Alexandru Ioan Cuza University of Iași, Iași, Romania; b Laboratory of Human Molecular Genetics, Faculty of Medicine of Sfax, University of Sfax, Sfax, Tunisia; SIPBS, University of Strathclyde

## Abstract

Paenarthrobacter nicotinovorans is a soil bacterium that uses the pyridine pathway to degrade nicotine. The genome of strain ATCC 49919 is composed of a ~4.3-Mbp chromosome and a ~165-kbp plasmid. The second strain, termed here *nic-*, is a cured derivative lacking the plasmid and not able to degrade nicotine.

## ANNOUNCEMENT

The Paenarthrobacter nicotinovorans nicotine degradation pathway, a potential renewable source of green chemicals (1), is encoded by the megaplasmid pAO1 (2). This pathway is also described in other nicotine-degrading microorganisms (NDMs) (3). To provide a needed NDM reference genome for further omics studies, we report here the complete genome sequences of *P. nicotinovorans* ATCC 49919 and a cured derivative unable to degrade nicotine, termed *P. nicotinovorans nic*-.

The strains, which had been previously isolated (4, 5), were a kind gift from Roderich Brandsch (ZBMZ, Albert Ludwig University of Freiburg), preserved at −80°C and grown on citrate medium supplemented with 0.05% nicotine (6). Log-phase cells were collected, washed, and treated with lysozyme (10 μg mL^−1^). For extracting DNA for short- and long-read sequencing, the DNeasy UltraClean microbial kit (Qiagen, Germany) and MagAttract HMW DNA purification kit (Qiagen), respectively, were used. The high-molecular-weight (HMW) DNA was further size selected using magnetic beads (AMPure XP, Beckman Coulter, USA) to a DNA ratio of 1.5:1 (7, 8).

A short-read sequencing library containing fragments of 470 bp was generated using the Illumina TruSeq DNA PCR-free kit (350-bp insert size; Illumina, USA) following standard procedures. Paired-end sequencing was performed using the NovaSeq 6000 system (Illumina). The reads were checked using FastQC v.0.11.9 (9) and filtered using fastp v.0.21.0 (7). A long-read sequencing library was generated using the rapid sequencing kit (ONT, UK) and sequenced on a MinION Mk1B (MIN-101B) device, using either the FLO-MINSP6 R9.4.1 or FLO-FLG001 R9.4.1 flow cells. The raw data were acquired using MinKNOW v.21.10.1, and high-accuracy basecalling was conducted using Guppy v.5.0.16. The output fastq files were checked using LongQC v.1.2.0c (10).

After filtering, >99% of the Illumina reads had Q scores of >30 and an average Phred score/read of 37. For the MinION reads, 87% of bases had Q scores of >7 and a mean quality value (QV)/read score of >7. For more descriptive statistics of the sequencing data, please see Table 1. Hybrid genome assembly was performed using Unicycler v.0.4.9 (7). Overlapping sequences at contig ends were removed so that each contig’s sequence led directly into its neighbors. The genomes were rotated to start with *dnaA* on the forward strand, assessed for completeness and contamination using CheckM v.1.0.9 (11), and uploaded to the NCBI PGAP v.5.3 (12) for automatic annotation. Searches against the 16S rRNA (*Bacteria* and *Archaea*) database from NCBI (accessed 1 March 2022) using BLASTN v.2.11.0 and rRNA sequences from both genomes indicated 98.2% identity with *P. nicotinovorans* strain DSM 420 (GenBank accession number NR_026194.1). For all software, default parameters were used.

**TABLE 1:** Basic sequencing metrics

Strain (BioSample accession no.)	Sequencing technology^*a*^	Raw data				Filtered data			
		SRA accession no.	Size (kbp)	No. of reads	GC (%)	Size (kbp)	No. of reads	Read length data (bp)	GC (%)
P. nicotinovorans ATCC 49919 (SAMN17383832)	SRS/Illumina	SRR13483967	2,091,873	13,853,462	63.05	1,934,725	12,812,750	150 (read length)	63.10
		SRR13483966	2,137,039	14,152,578	63.00	1,966,789	13,025,092		63.82
		SRR13483965	2,139,192	14,166,832	63.03	1,944,165	12,875,262		63.12
		SRR17074807	2,080,033	13,775,050	63.14	2,021,849	13,389,726		63.12
		SRR17074806	2,078,919	13,767,678	63.23	2,010,658	13,315,616		63.17
		SRR17074805	2,116,302	14,015,248	63.14	2,054,871	13,608,418		63.13
	LRS/MinION	SRR17083591	3,373,804	288,121	62.70			11,709.6 (mean), 21,146 (*N*_50_)	
	Total		16,017,162	84,018,969		15,306,861	79,314,985		
	Mean				63.04				63.17
P. nicotinovorans *nic-* (SAMN23721560)	SRS/Illumina	SRR17187325	2,083,609	13,798,734	63.36	2,020,264	13,379,230	150 (read length)	63.33
		SRR17187324	2,128,302	14,094,714	63.35	2,056,827	13,621,370		63.34
		SRR17187323	2,087,609	13,825,226	63.33	2,017,195	13,358,906		63.29
	LRS/MinION	SRR17187322	524,233	58,746	62.83			8,923.7 (mean), 16,496 (*N*_50_)	
	Total		6,823,753	41,777,420		6,618,519	40,418,252		
	Mean				63.22				63.20

a SRS, short-read sequencing; LRS, long-read sequencing. In the case of SRS, multiple sequencing runs were performed for each sample using the same sequencing library. Each sequencing run was deposited as an SRA entry.

**FIG 1:**
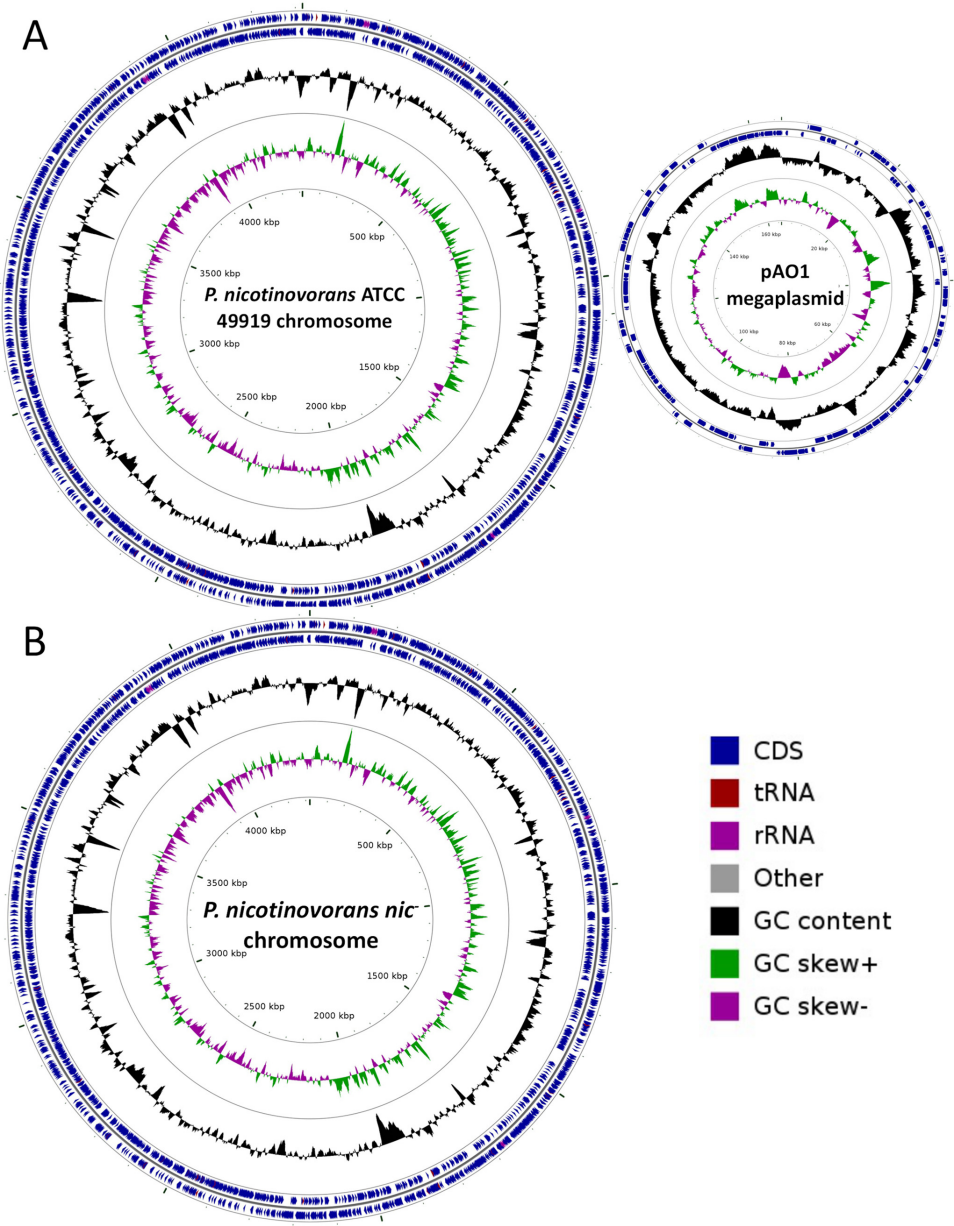
Circular maps of the genomes sequenced: (A) The *P. nicotinovorans* ATCC 49919 chromosome (left) and its megaplasmid, pAO1 (right); (B) the *P. nicotinovorans nic*- chromosome. The maps were generated using Circular Genome Viewer (CGView) v.1.14 (13).

The complete genome sequence of *P. nicotinovorans* ATCC 49919 was obtained and consists of two replicons: a 4,316,184-bp circular chromosome with an overall GC content of 63.2% and a 165,141-bp circular megaplasmid with an overall GC content of 59.7%. The genome assembly of *P. nicotinovorans nic*- indicated that this strain’s genome contains a single replicon of 4,323,902 bp, with the same overall GC content and number of noncoding RNAs (ncRNAs), transfer-messenger RNAs (tmRNAs), and ribosomal operons as the strain harboring the megaplasmid pAO1 (Fig. 1).

### Data availability.

The complete and functionally annotated genome sequences have been deposited at NCBI GenBank under the following accession numbers: CP089293 (*P. nicotinovorans* ATCC 49919 chromosome); CP089294 (*P. nicotinovorans* ATCC 49919 megaplasmid pAO1); and CP089515 (*P. nicotinovorans nic*- chromosome). The sequencing data are available under NCBI BioProject accession number PRJNA693273 and under the BioSample and SRA accession numbers listed in Table 1.
